# The emerging sub-genotype C2 of *Coxsackievirus*A10 Associated with Hand, Foot and Mouth Disease extensively circulating in mainland of China

**DOI:** 10.1038/s41598-018-31616-x

**Published:** 2018-09-06

**Authors:** Tianjiao Ji, Yue Guo, Wei Huang, Yong Shi, Yi Xu, Wenbin Tong, Wenqing Yao, Zhaolin Tan, Hanri Zeng, Jiangtao Ma, Hua Zhao, Taoli Han, Yong Zhang, Dongmei Yan, Qian Yang, Shuangli Zhu, Yan Zhang, Wenbo Xu

**Affiliations:** 1 Ministry of Health Key Laboratory for Medical Virology, National Institute for Viral Disease Control and Prevention, Chinese Center for Disease Control and Prevention, Beijing, People’s Republic of China; 2 Hunan Center for Disease Control and Prevention, Changsha, Hunan Province People’s Republic of China; 3 Jiangxi Center for Disease Control and Prevention, Nanchang, Jiangxi Province People’s Republic of China; 4 Shaanxi Center for Disease Control and Prevention, Xi’an, Shaanxi Province People’s Republic of China; 5 Sichuan Center for Disease Control and Prevention, Chengdu, Sichuan Province People’s Republic of China; 6 Liaoning Center for Disease Control and Prevention, Shenyang, Liaoning Province People’s Republic of China; 7 Tianjin municipal Center for Disease Control and Prevention, Tianjin municipal, People’s Republic of China; 8 Guangdong Center for Disease Control and Prevention, Guangzhou, Guangdong Province People’s Republic of China; 9 Ningxia Center for Disease Control and Prevention, Yinchuan, Ningxia Province People’s Republic of China; 10 Chongqing Center for Disease Control and Prevention, Chongqing municipal, People’s Republic of China

## Abstract

Coxsackievirus A10 (CV-A10) associated with Hand, foot, and mouth disease (HFMD) cases emerged increasingly in recent years. In this study, the samples from nation-wide HFMD surveillance, including 27 out of 31 provinces in China were investigated, and the continuous and extensive virological surveillance, covered 13 years, were conducted to provide a comprehensive molecular characterization analysis of CV-A10. 855 CV-A10 viruses (33 severe cases included), were isolated from HFMD children patients during 2009 to 2016 in China. 164 representative sequences from these *viruses*, together with 117 CV-A10 sequences downloaded from GenBank based on entire VP1 were recruited in this study. Two new genotypes (F and G) and two sub-genotypes (C1 and C2) were identified. *Among 264 Chinese sequences, 9 of them were genotype B, 8 of them were C1, and the other (247) were C2*, the predominant sub-genotype in China since 2012. Chinese C2 viruses showed obvious temporal characteristics and can be divided into 3 clusters (cluster 1~3). Cluster 3 viruses was circulating extensively during 2014 and 2016 with more severe cases. *It is very necessary and important to continuously conduct the extensive virological surveillance for CV-A10, and further evolutionary studies will provide more evidence on its evolution and virulence*.

## Introduction

Hand, foot and mouth disease (HFMD) is a common contagious disease among children. E*nterovirus* (EV), especially *enterovirus*A71 (EV-A71)^1^ and *coxsackievirus* A16 (CV-A16)^2^, are the etiological agents involved in HFMD.

HFMD has been listed as the 38th notifiable disease in China since 2008. Following that, an extensive three-level HFMD surveillance laboratory network including 1 national lab, 31 province labs and 331 prefectural labs was established. Besides of EV-71 and CV-A16, CV-A10 has been increasingly associated with sporadic HFMD cases and outbreak events globally, compared with other HEV. A large outbreak of HFMD associated with CV-A10 and *coxsackievirus* A6 (CV-A6) were reported in France in 2010^3^. CV-A10 and *coxsackievirus* B1 (CV-B1) were detected as a co-infection in a childcare center in Spain, where an onychomadesis after HFMD occurred in 2008^4,5^. In the same year, CV-A10 was reported as the most prevalent virus causing the outbreak of HFMD in the Singapore^6^. In mainland of China, following EV-71 and CV-A16, CV-A10 was the third most common virus detected in HFMD during 2009–2011^7^. Most diseases associated with CV-A10 were wild and self-limiting, but there also had severe^8^ and death cases^9^ been reported in domestic and overseas.

Several studies on CV-A10 genotyping were based on 5′-UTR^10^, VP4^11^, or partial VP1^3,4,12,13^. *However, VP1 plays a critical role in mediating binding receptor and the complete VP1 has been used widely to identify EV serotypes*^14–18^ *as its coding region contains many important neutralizing antigenic sites. According to another recent study, the cell surface molecule KREMEN1 was verified as an entry receptor for CV-A10*^19^*, KREMEN1 overexpression enhances CV-A10 binding to the cell surface and increases susceptibility to infection, indicating that KREMEN1 is a rate-limiting factor for CV-A10 infection*.

Although CV-A10 occupied certain proportion in HFMD pathogenic spectrum, its prevalence, genetic character and the criterion for genotyping are still not clear. In this study, the samples from nation-wide HFMD surveillance, including 27 out of 31 provinces in China were investigated, and continuous and extensive virological surveillance were conducted to cover more than 13 years to provide a comprehensive epidemiological and molecular characterization analysis of CV-A10, and to establish the standardization for genotyping.

## Results

### Temporal and geographic distribution of CV-A10 isolates circulated in China from 2004 to 2016

A total of 15361 strains were identified as positive for EV, including 7360 EV-A71, 4667 CV-A16, 3330 other EV (non EV-A71&CV-A16). Among the other EV, there were 855 CV-A10 viruses (Table 1). *The number of CV-A10 cases were increasing, and the proportion of CV-A10 associated with severe cases were on the rise as well during 2012 and 2016, as shown in* Table 1.

**Table 1:** Temporal and serotype distribution of EV isolates circulated in China from 2008 to 2016.

Year	All	EV-A71	CV-A16	Other EV	CV-A10	CV-A10/ Other EV	Severe cases	Severe case caused by other EV(proportion)	Severe case caused by CV-A10 (proportion)
2008	389	236	94	59	0	0.00%	8	0(0.00%)	0(0.00%)
2009	601	399	130	72	11	15.28%	84	9(10.71%)	0(0.00%)
2010	1644	1026	475	143	38	26.57%	379	37(9.76%)	7(1.79%)
2011	1204	805	285	114	17	14.78%	154	9(5.84%)	1(0.62%)
2012	1288	625	518	145	68	46.90%	175	17(9.71%)	1(0.52%)
2013	2490	1022	541	927	84	9.05%	152	65(42.76%)	5(2.76%)
2014	2618	1203	1006	409	170	41.56%	224	23(10.27%)	8(3.45%)
2015	2598	1069	850	679	127	18.68%	153	17(11.11%)	6(3.53%)
2016	2529	975	768	782	340	43.48%	110	20(18.18%)	5(4.55%)
Total	15361	7360	4667	3330	855	25.68%	1439	197(13.69%)	33(2.29%)

**Figure 1:**
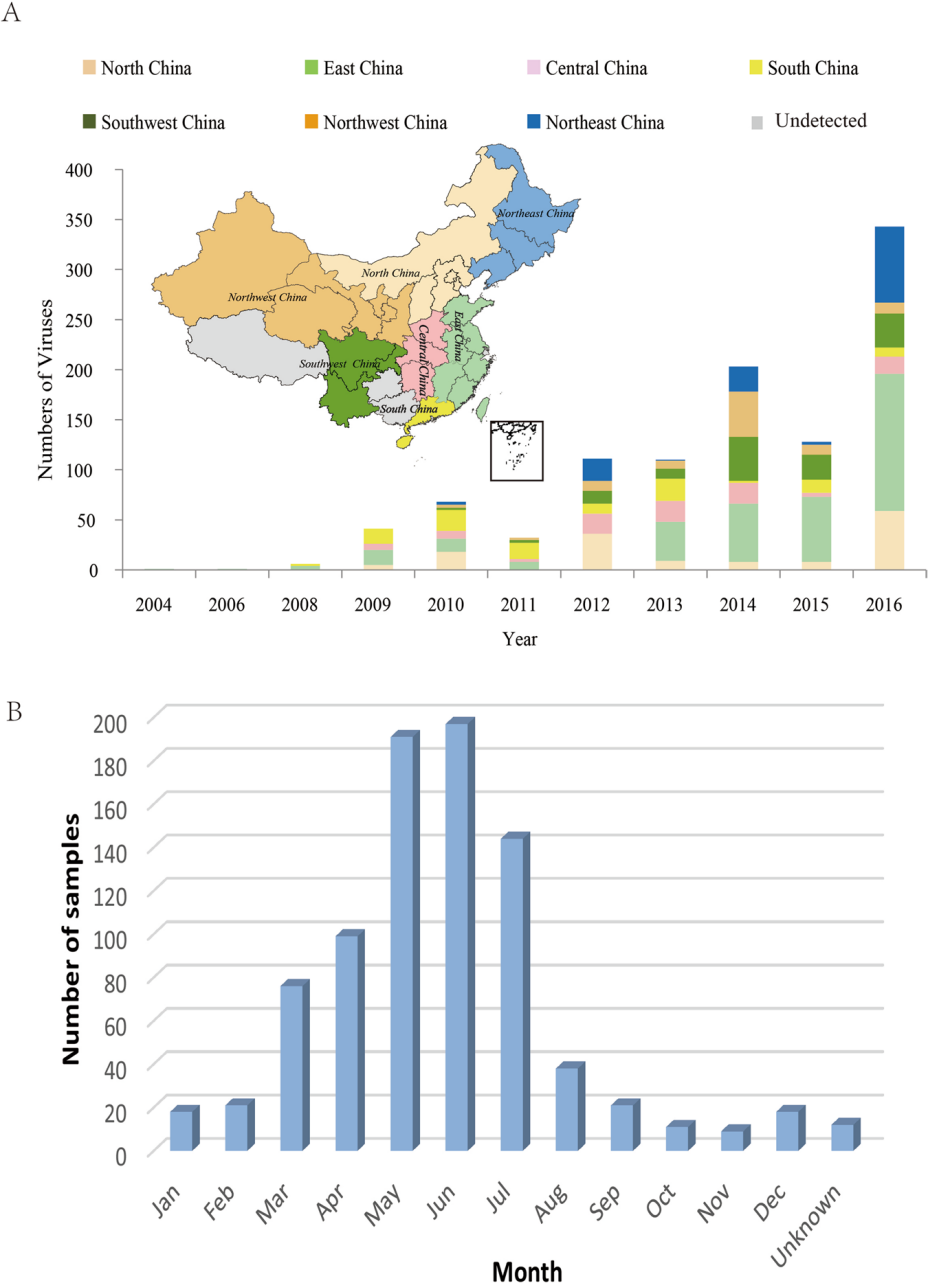
(**A**) Temporal and geographic distribution of 1044 Chinese CV-A10 viruses during 2004–2016. (**B**) Number of cases of HFMD by month of illness onset.

855 CV-A10 viruses from 26 out of 31 provinces obtained in this study, together with 189 Chinese CV-A10 sequences downloaded from GenBank during 2004–2016 were included in this study for further analysis. (Fig. 1A, Supplementary Table S1). *The data showed that CV-A10 has been extensively circulating around the mainland of China since 2012, covered 7 geographic regions and 27 out of 31 provinces of mainland of China (one additional province involved in GenBank) during 2004–2016*. While before 2012, CV-A10 was distributing in limited regions and provinces with much less number of cases.

Thirty-three out of 855 cases were diagnosed as severe cases: patients with neurological complications or cardiopulmonary complications according to the National Guideline for Diagnosis and Management of HFMD cases issued by National Health and Family Planning Commission of China in 2010 (http://www.nhfpc.gov.cn/zwgkzt/wsbysj/201004/46884.shtml). Most of patients (716/855, 83.84%) were 1~4 years old, the median age of these patients was 2.00 years (range 0.1–24 years), while 95.12% of severe cases were less than 3 years old. Nearly 3/4 (631/855, 73.81%) of cases occurred during April and July (Fig. 1B), when was spring and summer time in China.

### Seven genotypes of CV-A10 were assigned based on entire VP1

There were 284 VP1 sequences available from GenBank before November 15^th^, 2017. All these VP1 sequences were from 9 countries during 1950 and 2016, including the prototype virus Kowalik strain. The representative sequences were selected to include the sequences covering all the location/country/provinces and time range, meanwhile the sequences with high homologies or with significant errors in sequences, were not included in this study. In all, total 281sequences were included to be performed the phylogenetic tree, including 164 out of 855 VP1 sequences in this study and 117 out of 284 sequences from GenBank.

**Figure 2:**
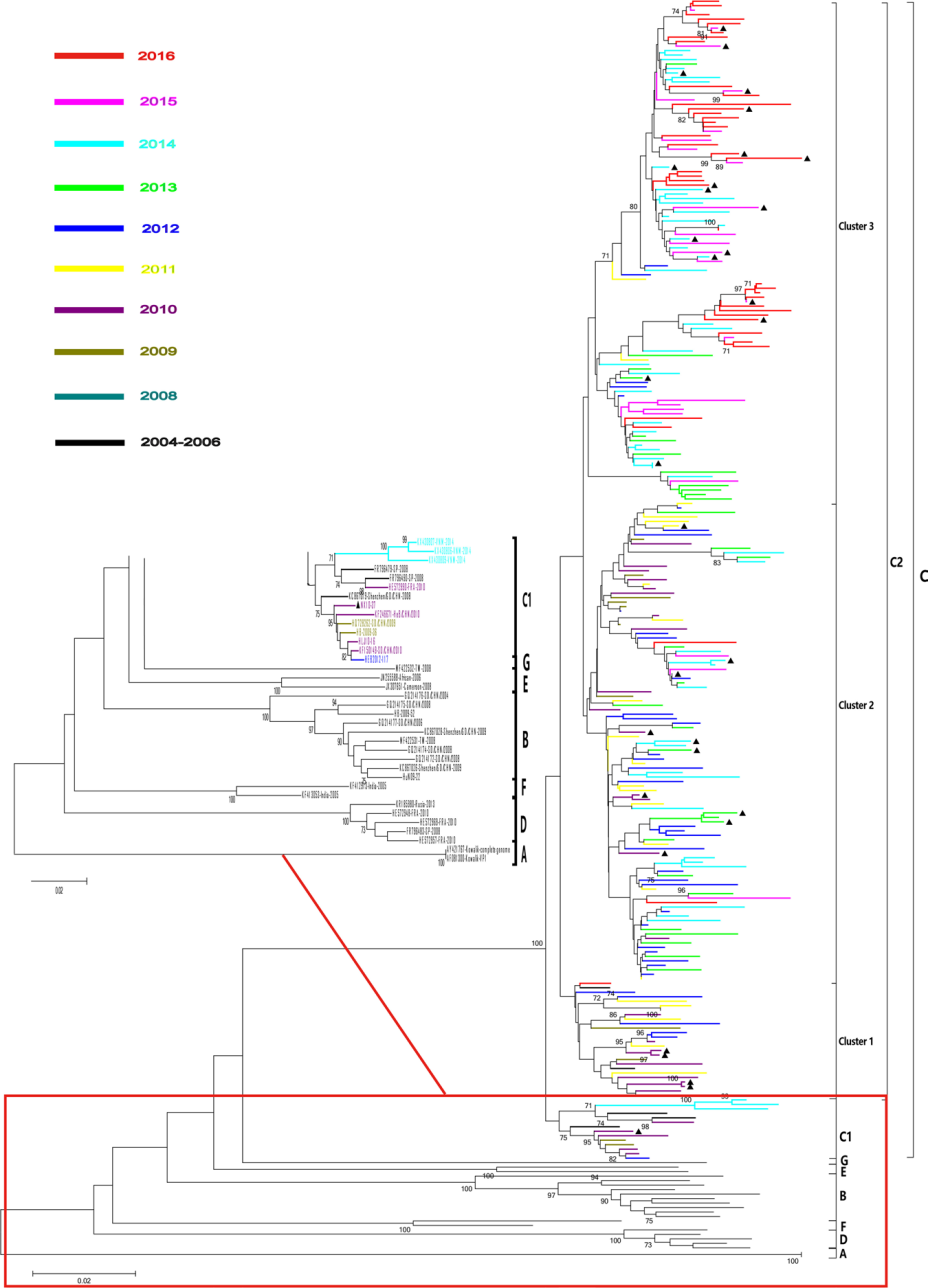
Phylogenetic dendrogram was drawn on bases of the 894-nt sequences for 281 representative CV-A10 isolates from 2004–2016. The Striains that isolated from different years were represent by different color according to the legend. Solid triangle indicated the severe cases. Sequences downloaded from GenBank was list in Supplementary Table S1.

**Figure 3:**
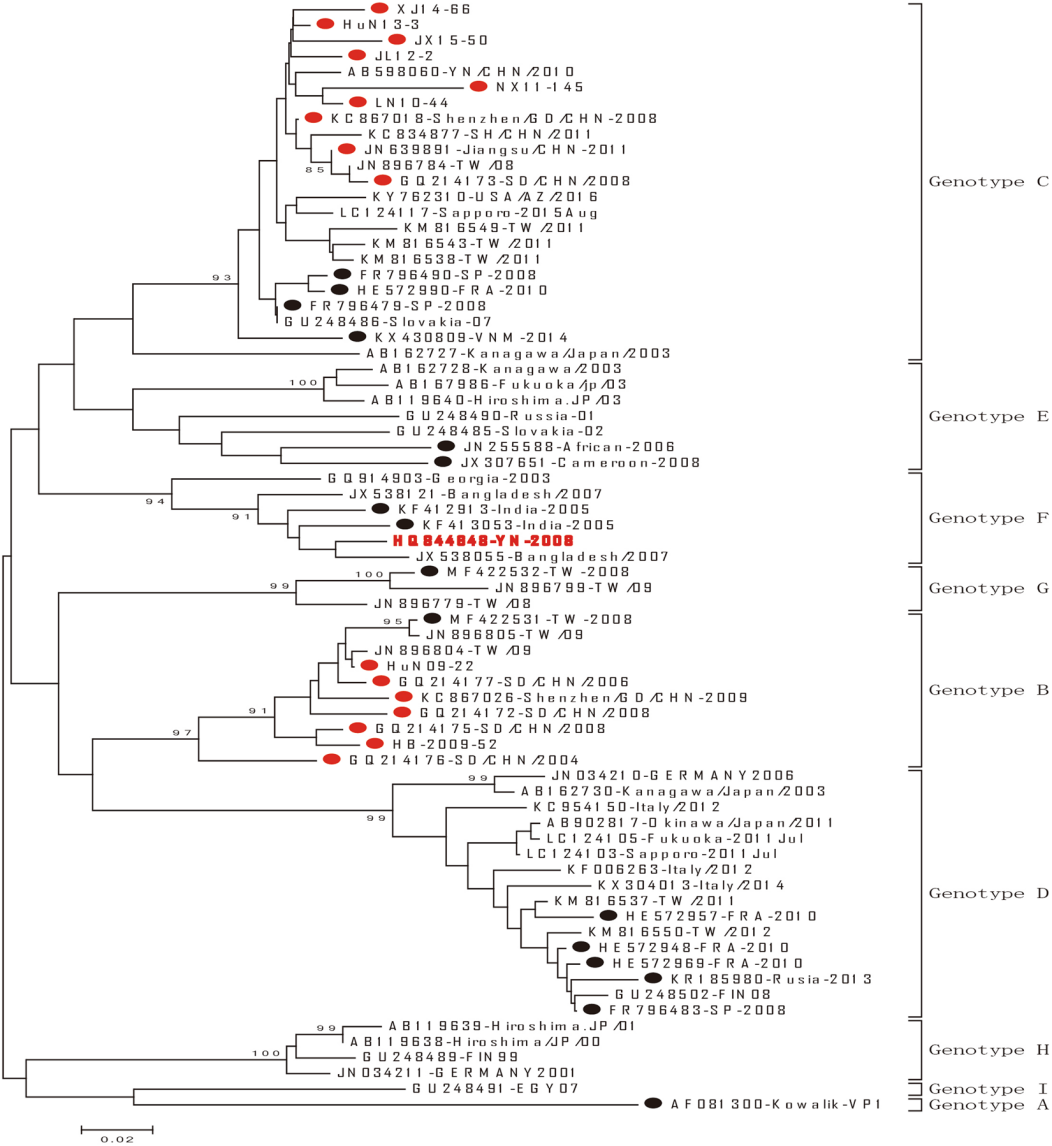
Phylogenetic analyses based on all the available partial VP1 (239 bp on the 5′ of VP1 region) of CV-A10 in the world. Representative sequences of entire VP1 selected from different genotypes and time in the world were labeled with solid circle, of which Chinese sequences were labeled with solid red circles.

A phylogenetic tree was constructed based on these representative viruses of CV-A10, and the sequences were assigned to seven genotypes (A, B, C, D, E, F and G) with at least 14.97% nucleotide divergence between different genotypes (Fig. 2). And two new genotypes (F and G) were assigned in this study, together with previously assigned genotypes A-E^18,20^. *Genotype F was consisted of two Indian sequences in 2005, although, these two sequences were not full length of VP1, with 7 bases or 22 bases deletion in 3′ terminal of VP1 region in individual Indian sequences, respectively*. One Taiwan China sequence isolated in 2008 was grouped separately, assigned genotype G, and it seems that it circulated only in Taiwan China (Fig. 3). Pairwise nucleotide and amino acid sequences identities in VP1 region between different genotype of viruses in this study were showed in following table (Table 2).

**Table 2:** Pairwise nucleotide and amino acid sequences identities in VP1 region between different genotype of viruses.

Genotype	% identity in VP1		Isolated country/year	Notes
	(Nucleotide sequences)	(Amino acid sequences)		
A	75.43~77.89	90.88~93.33	America/1950	Prototype strain
B	76.14~83.79	91.37~95.19	China/2004–2009	Ceased genotype
C	76.43~85.03	92.97~96.11	China, Spain, Vietnam, France	Predominant genotype in China
D	75.89~82.17	91.44~94.25	France/2010, Spain/2008, Russia/2013	/
E	77.10~84.39	92.46~96.32	West Africa/2006, Cameroon/2008	/
F	77.57~82.99	93.33~96.32	Indian/2005	Newly named
G	75.43~85.03	90.88~94.96	Taiwan China/2008	Newly named

### Representative viruses of CV-A10 on partial VP1 in the world

As complete VP1 sequences of CV-A10 available were limited, in order to investigate CV-A10 virus’s origin and spread in the world, and to verify the genotypes on entire VP1, the representative viruses of CV-A10 on partial VP1 were selected and performed the phylogenetic analysis (Fig. 3). Based on 239 bps of VP1, we found that CV-A10 can be divided into 9 genotypes, with the mean group distance 14.5–24.6%. Compared with the entire VP1 region tree, those selective partial VP1 sequences were still in the same cluster, except for two potential genotypes (H and I) isolated in earlier year but been ignored. Genotype H was composed of sequences before 2001 that emerged in Japan, Finland and Germany, and genotype I was only composed with one Egypt strain of 2007, which was 18.70% nucleotide difference with the prototype strain Kowalik.

It seems that viruses circulated in Asia and Europe during recent years were quite different: European viruses (Germany 2006, Finland 2008, Spain 2008, France 2010, Italy 2012–2014 and Russia 2013) were circumscribed in genotype D, while Asian viruses were more diversity and distributing in mainland of China -B and C, Indian- F, Taiwan China-B, C, D and G, Japan-C, D, E and H.

Besides, we found one Yunnan strain (HQ844848-YN-2008) in the border areas with Myanmar has the closest relative to the Indian viruses of genotype C, which was probably as an imported case.

### Two genotypes of CV-A10 had been persistently circulated in China from 2004 to 2016

There had been two genotypes circulating in China: genotype B had been active during 2004–2009, and genotype C has been evolved as the absolutely predominant genotype since 2009, and can be further divided into C1 and C2 sub-genotypes. Sub-genotype C1 was circulating during 2008–2012, while C2 was the predominant sub-genotype after 2010. The nucleotide divergence between sub-genotypes C1 and C2 was 5.8%. All the other Chinese viruses grouped in C2 and can be categorized into 3 clusters: C2-cluster1, C2-cluster2, and C2-cluster3.

The group mean distance among these 3clusters were range from 3.7% to 6.2%. Chinese viruses showed obvious temporal characteristics (Fig. 3A): viruses from 2008–2010 were mainly groupeded in cluster 1 and cluster 2, while most of sequences from 2015–2016 were grouped into cluster 3. Cluster 2 included 2009–2016 sequences, while 2011–2014 sequences were the primary members.

*We have compared the nucleotide and amino acid mutations among the sub-genotype C1, C2, and C2 cluster 1~cluster3, described in the* Table 3 *below. Compared with C1, cluster 1 and cluster 2 of C2, cluster3 of C2 had two major significant mutation in VP1 region (on nucleotide site of 68 and 847, respectively) and resulted in amino acid variations. For example, there were 63/107 (58.88%) sequences have mutation from glycine (G) to valine (V) on nucleotide site 68 in Cluster3 viruses while 4/14(28.57%), 0/16(0.00%), 7/115 (6.09%) in C1, cluster 1 and cluster2, respectively. And similar differences between C1, cluster 1~2 of C2 and cluster3 of C2 were observed in nucleotide site 847 as well* (Table 3)*. In addition, one synonymous mutation on nucleotide site 813 was also observed: most of sequences from C2 cluster 2 and cluster 3 had the mutation of cysteine (C) to glycine (G), while the C1 and cluster 1 were barely found. Homologous comparison analysis on nucleotide sequences showed that there was no difference between mild cases and severe cases in amino acid coding region. However, we found all the severe cases located in C2 except for one Ningxia strain in 2010*, and nearly half of severe viruses (17/33) located in cluster3, which was continuous circulating during 2014 and 2016.

**Table 3:** The Nucleotide and Amino acid mutations among the su-genotype C1, cluster 1, cluster 2 and cluster 3 of sub-genotype C2 in VP1 region.

Sub-genotype/ Cluster		Total number of sequences	Nucleotide	Amino acid	Nucleotide	Amino acid	Nucleotide	Amino acid
			(68/894)	(23/298)	(813/894)	(271/298)	(847/894)	(283/298)
			G → T	G → V	C → G	L	A → G	I → V
C1		14	4(28.57%)*		0*		1(7.14%)*	
C2	Cluster-1	16	0*		1(6.25%)*		0*	
	Cluster-2	115	7(6.09%)*		111(96.52%)*		4(3.48%)*	
Cluster-3		107	63(58.88%)*		95(88.79%)*		57(53.27%)*	

Note: * indicated the number and proportion of variation about nucleotide or amino acid.

The average evolutionary divergence of genotype C sequences between different time groups was calculated using MEGA 5.03 with p-distance model (Table 4). The results revealed that the nucleotide divergences within individual year from 2008–2016 were increasing gradually, except for 2009, as limited viruses available in 2009. Analysis of all these viruses did not show obvious regional distribution characteristics (Fig. 3B).

**Table 4:** The average evolutionary divergence of genotype C sequences between different time groups.

Year	Year/Divergence								
	2008	2009	2010	2011	2012	2013	2014	2015	2016
2008	**0.0293**	0.012	0.010	0.010	0.011	0.013	0.014	0.016	0.014
2009	*0.042*	**0.0475**	0.014	0.014	0.016	0.017	0.018	0.020	0.018
2010	*0.034*	*0.040*	**0.0341**	0.012	0.013	0.014	0.015	0.017	0.016
2011	*0.037*	*0.040*	*0.033*	**0.0317**	0.011	0.012	0.014	0.016	0.015
2012	*0.040*	*0.043*	*0.036*	*0.033*	**0.0334**	0.012	0.013	0.016	0.015
2013	*0.044*	*0.047*	*0.040*	*0.037*	*0.037*	**0.0401**	0.015	0.017	0.017
2014	*0.046*	*0.048*	*0.042*	*0.039*	*0.040*	*0.042*	**0.0416**	0.016	0.016
2015	*0.048*	*0.050*	*0.045*	*0.042*	*0.043*	*0.045*	*0.042*	**0.0419**	0.017
2016	*0.050*	*0.054*	*0.048*	*0.046*	*0.048*	*0.050*	*0.047*	*0.047*	**0.0501**

Note: The left-lower data in italics are nucleotide diversity. The right-upper data in normal font are deduced amino acid sequence diversity. Mean nucleotide diversities within years (this study) are marked in the bold underline.

## Discussion

HFMD is a common disease among children, especially for children below five years. Although EV-71 and CV-A16 were the predominant pathogens caused HFMD in recent years, the proportion of other EV has been increasing^7,20–22^, among which, CV-A10 was reported to be associated with sporadic HFMD cases and outbreak events in several countries^3,5,6,12^ and topped the list of other EV in many provinces of China^23,24^. In this study, we found an apparently increasing proportion of CV-A10 among HFMD cases in mainland of China since during 2012 and 2016, except for 2013 and 2015, when CV-A6 was responsible for most of HFMD outbreaks in China^25–27^. This reveals that CV-A10 is gradually becoming one of the most frequent EV serotypes during the epidemic interval of EV-71 and CV-A16. As most of the studies were performed based on province level with a small sample size. This study, we collected the nationwide representative samples and covered over a 13 years’ time span in order to provide a comprehensive epidemiology and molecular characterization analysis of CV-A10.

All age groups are susceptible to be infected with CV-A10, but most cases have occurred in children below 4 years old (with median age of 2), which was similar with the infection occurred in Spain^4,5^, France^3^ and Singapore^6,28^. However, the median age was 6 years old in the HFMD outbreak caused by CV-A6 and CV-A10 of Finland^12^, and it was initially ignored because of most cases were adult infections, which suggested that a low herd immunity to these viruses. Most of reports about CV-A10 in China occurred mainly in spring and summer^10,29–32^, like the EV infection, but the 84% of HMFD cases in Finland were reported in October –December (autumn and winter).

The first strain of CV-A10 was isolated in New York in 1950^33^, prototype of CV-A10, Kowalik strain. Since then, CV-A10 was uncommon virus in Europe and United States^34–38^ until several outbreaks was reported in Europe during 2008–2010. China national notifiable disease reported system showed that the proportion of other EV associated with HFMD was increasing in recent years^22^. In this study, we further identified the CV-A10 was the major pathogen of other EV responsible for HFMD in China. The number of CV-A10 circulating in China was becoming more and more prevalent (more patients and more provinces involved) and had a trend of increasing severe cases proportion from 2012–2016 (Table 1 and Fig. 1). This is a very important pathogenic information to guide the EV71 vaccination^39–41^ and HFMD control and treatment.

Seven genotypes were assigned based on entire VP1 in this study, which was consistent with other studies^18,20,42^ except for two new genotypes F and G. In addition, we rebuilt a phylogenetic tree on partial VP1 to investigate CV-A10 virus’s origin and spread in the world, and to verify the genotype classification based on method of complete VP1. In general, the genotyping results were consistent with each other, and two potential genotypes H and I were identified in separate group according to partial VP1. The comprehensive search from available public database showed H and I genotype viruses were obtained only from Japan, Finland, Germany and Egypt before 2007, and no more viruses were detected after that. Both of these genotypes might be disappeared after 2007, or they could not be detected because of surveillance gap. However, it is necessary to obtain the VP1 complete sequences of these suspicious genotype viruses to verify its genotyping classification and to better understand its spread in the world. As VP1 complete coding region contains many important neutralizing antigenic sites and there were generally consistent classification of genotyping between two sequencing window, we recommended VP1 to be as the standard target sequencing window for CV-A10.

According to both complete VP1 and partial VP1 sequences, we found the similar situation: different genotype viruses have different geographic distribution pattern. Genotype B and G were mainly circulating in Chinese mainland and its surrounding areas, such as Taiwan China. While *most of Chinese viruses*, European viruses and Indian surrounding viruses, which solely clustered in genotype C, D, and F, respectively, showing geographic distribution circumscribed characterization. This is similar to other EV, like EV-A71^1^, but different with respiratory viruses such as measles^43,44^ and human respiratory syncytial viruses^45,46^, which spread globally and different genotypes distributed cross over with each other in phylogenetic tree, not much geographic distribution circumscribed characterization.

**Figure 4:**
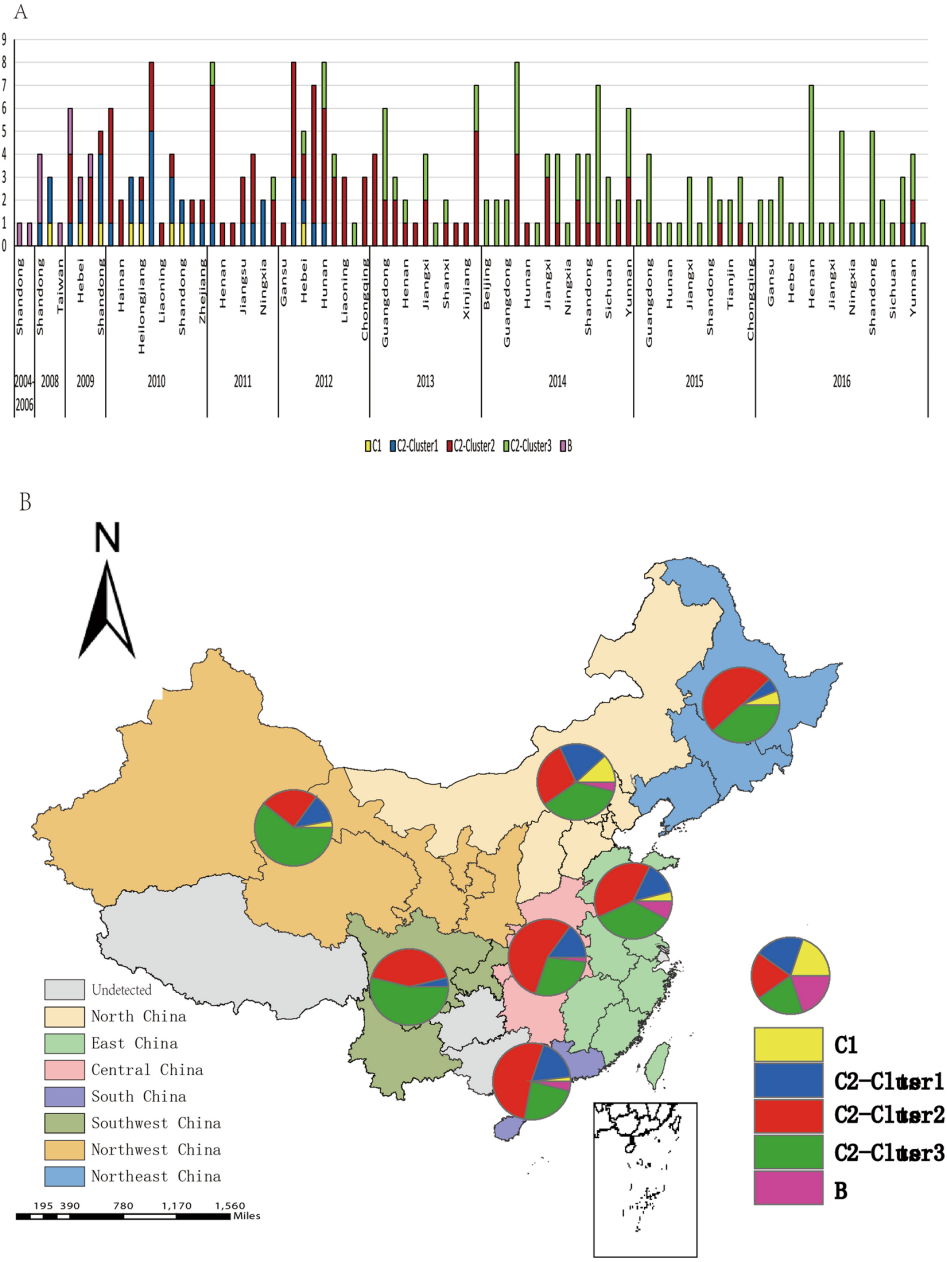
(**A**) Yearly and geographic distribution of genotypes and clusters of CV-A10 in china from 2004 to 2016. Different genotypes and clusters were colored according to the legend. (**B**) The geographic distribution of HFMD associated CV-A10 viruses in 7 representative regions of China. Different regions and numbers were colored according to the legend (Taiwan China sequences were not included). The free map was required from ArcGis.

There was an obvious genotype shift occurred in CV-A10 epidemic during 2004–2016 in mainland of China. Genotype B had been primary genotype circulating during 2004–2009, following by genotype C becoming predominant genotype during 2010–2016. It might not rule out the undetected genotype C before 2008, because of HFMD surveillance was initiated. Later on, based on the extensive and continuous HFMD surveillance, we concluded genotype B had disappeared completely. Similarly, sub-genotype C1 was mainly circulating during 2008–2012 and limited in few areas, but C2 gradually replaced C1 and became the predominant sub-genotype especially after 2010, which was similar to the alternation between C4a and C4b of EV-71 in China^15,17^. As the spread of sub-genotype C2 viruses, it has been evoluted and divided into 3 clusters. Viruses that isolated between 2008~2013 usually located in the cluster 1 and cluster 2, and 2014–2016 viruses were clustered in cluster 3. Comparing with cluster 1 and 2 viruses, cluster 3 viruses distributed in much more province with more prevalence during 2014 and 2016 (Fig. 4).

*According to the mutations analysis among these sub-genotypes and clusters, we found that C2-cluster3 had three significant mutations and resulted in two nonsynonymous mutations and one synonymous mutation in VP1 region. Studies have shown that VP1 plays a critical role in mediating binding receptor, these mutations might cause some changes on the structure and function of VP1 protein, which might make the viruses having stronger transmissibility, infectivity and virulence*.

The average evolutionary divergence of genotype C sequences between different year revealed that the nucleotide divergences within individual year from 2008–2016 were increasing gradually (Table 3), and this indicated that CV-A10 evolved constantly over years, its gene polymorphism gradually increased in these years. Based on Bayesian theory^17^, the scale of the pandemic was associated with the gene polymorphism: as the gene polymorphism increased, so do the scale of the pandemic. This is consistent with the findings in this study.

Most diseases associated with CV-A10 were mild and self-limiting, but the data from Singapore HFMD outbreak of in 2008 showed: comparing with EV-71, the virulence of CV-A10 and CV-A6 might be lower, but their transmissibility was stronger^6^. In the study of Cao *et al*., CV-A10 was demonstrated to be associated with severe complications defined by the same criteria, although with less effect than EV-71^7^. In this study, we found that the proportion of severe cases caused by CV-A10 had been gradually increasing since 2012. In addition, nearly half of severe viruses (17/33) located in cluster3.

All these evidence indicated C2-cluster3 viruses might have been evoluted with stronger transmissibility and virulence during the fitness with the immunity of host after persistent circulation in population. It is very necessary and very important to conduct the continuous and extensive virological surveillance for CV-A10, and further evolutionary studies, to better understand its evolution, transmissibility and virulence, will provide more evidence based scientific data to guide disease control and treatment.

## Methods

### Sample collection and virus isolation

*Stool, throat swabs, or nasal swabs from HFMD patients were collected according to standard protocols from national HFMD guideline (*http://www.gov.cn/gzdt/2009-06/04/content_1332078.htm*). All these samples were primarily tested by the commercial real-time RT-PCR (Shuoshi Biotech, Jiangsu, China), and all CV-A10 positive samples were incubated into RD or HEp-2 cell lines in provincial laboratories. The infected cells were cultured for at least 2 passages until cytopathic effect occurred, and then, the isolates were harvested and shipped to national reference laboratory for sequencing*.

### VP1 Amplification and RT-PCR amplification

Viral RNA was extracted from the virus culture using QIAamp viral RNA mini kit (Qiagen, Valencia, CA) according to the manufacture’s recommended procedure. Specific primers were designed to amplify the entire VP1 gene using the Primer 3.0 (http://bioinfo.ut.ee/primer3-0.4.0/). CV-A10-F: 5′-GAAACCCCTGGAGAGGCATA- 3′ (nucleotides 2329–2348, relative to strain CV-A10/Kowalik), CV-A10-R: 5′-TCGTGAGCTATCTTCCCACA-3′ (nucleotides 3445–3426, relative to strain CV-A10/Kowalik).

One step RT-PCR was performed using the One Step PT-PCR kit Ver.2 (TaKaRa, #RR057A). The reaction system as followed: 3 μl of viral RNA was added to reaction mixture (total volume 25 μl), containing 12.5 μl2 × Buffer, 7.5 μldeionized H_2_O, 1 μl Enzyme Mix, and 0.5 μl each of the specific primers. PCR profile were 50 °C for 30 min, then 94 °C for 3 min; 32 cycles at 94 °C for 30 s, 50 °C for 30 s and 72 °C for 1 min and 20 s; and final extension step at 72 °C for 10 min. The products were analyzed by 1.5% agarose gel electrophoresis, and positive products were purified using the QIAquick Gel extraction kit (Qiagen, Valencia, CA). All the amplicons were sequenced by using both upper and down primers on an ABI Prism 3100 genetic analyzer^29^ (Applied Biosystems, Hitachi, Japan).

### Phylogenetic analysis

The entire VP1or partial VP1 sequences of the CV-A10 viruses were aligned by the MEGA (Version 5.03) program (Sudhir Kumar, Arizona State University, Tempe, AZ) with all of available sequences downloaded from GenBank (Supplementary Table S2). Phylogenetic analysis using neighbor joining (NJ) and maximum likelihood (ML) was performed. A phylogenetic tree was constructed with the Kimura-2 parameter evolutionary models, and the reliability of it was tested by 1000 bootstrap replicates. Bootstrap values greater than 80% were considered statistically significant for grouping.

### Nucleotide Accession Number

The entireVP1 nucleotide sequence that represent different year and genotype/cluster of this study were deposited in the GenBank database under the accession number KF999730– KF999786 and MG838781–MG838884. (Supplementary Table S3).

### Ethics Statement

This study did not involve human participants or human experimentation. Only specimen (stool samples, throat swab samples) collected from HFMD patients for public health purposes at the urging of the Ministry of Health, P. R. of China. Written informed consent for the use of their clinical samples was obtained from the parents of the children whose samples were analyzed. This study was approved by the second session of the Ethics Review Committee of the National Institute for Viral Disease Control and Prevention (NIVDC), Chinese Center for Disease Control and Prevention, all experimental protocols were approved by NIVDC, and the methods were carried out in accordance with the approved guidelines.

## Electronic supplementary material

Geographic distribution of 1044 Chinese CV-A10 strains over time

Reference strains of CV-A10 used for phylogenetic analysis from GenBank

The locality and time distribution of the CV-A10 VP1 sequences involved in this study

## References

[1] Zhang Y (2013). Complete genome analysis of the C4 subgenotype strains of enterovirus 71: predominant recombination C4 viruses persistently circulating in China for 14 years. Plos One.

[2] Chen X (2013). Molecular epidemiology of coxsackievirus A16: intratype and prevalent intertype recombination identified. Plos One.

[3] Mirand A (2012). Outbreak of hand, foot and mouth disease/herpangina associated with coxsackievirus A6 and A10 infections in 2010, France: a large citywide, prospective observational study. Clin Microbiol Infec.

[4] Bracho MA, González-Candelas F, Valero A, Córdoba J, Salazar A (2011). Enterovirus Co-infections and Onychomadesis after Hand, Foot, and Mouth Disease, Spain, 2008. Emerg Infect Dis.

[5] Davia JL (2011). Onychomadesis Outbreak in Valencia, Spain Associated with Hand, Foot, and Mouth Disease Caused by Enteroviruses. Pediatr Dermatol.

[6] Wu Y (2010). The largest outbreak of hand; foot and mouth disease in Singapore in 2008: The role of enterovirus 71 and coxsackievirus A strains. Int J Infect Dis.

[7] Lu QB (2012). Circulation of Coxsackievirus A10 and A6 in hand-foot-mouth disease in China, 2009–2011. Plos One.

[8] Chen M (2017). Severe hand, foot and mouth disease associated with Coxsackievirus A10 infections in Xiamen, China in 2015. J Clin Virol.

[9] Fuschino, M. E., Lamson, D. M., Rush, K., Carbone, L. S. & Taff, M. L. Detection of coxsackievirus A10 in multiple tissues of a fatal infant sepsis case. *J Clin Virol* **259** (2012). 10.1016/j.jcv.2011.12.011 22209288

[10] Guan H (2015). Etiology of Multiple Non-EV71 and Non-CVA16 Enteroviruses Associated with Hand, Foot and Mouth Disease in Jinan, China, 2009—June 2013. Plos One.

[11] Yamashita T, Ito M, Taniguchi A, Sakae K (2005). Prevalence of coxsackievirus A5, A6, and A10 in patients with herpangina in Aichi Prefecture, 2005. Jpn J Infect Dis.

[12] Blomqvist S (2010). Co-circulation of coxsackieviruses A6 and A10 in hand, foot and mouth disease outbreak in Finland. J Clin Virol.

[13] He Y (2013). Emergence, Circulation, and Spatiotemporal Phylogenetic Analysis of Coxsackievirus A6- and Coxsackievirus A10-Associated Hand, Foot, and Mouth Disease Infections from 2008 to 2012 in Shenzhen, China. J Clin Microbiol.

[14] Oberste MS, Maher K, Kilpatrick DR, Pallansch MA (1999). Molecular evolution of the human enteroviruses: correlation of serotype with VP1 sequence and application to picornavirus classification. J Virol.

[15] Zhang Y (2011). Emergence and transmission pathways of rapidly evolving evolutionary branch C4a strains of human enterovirus 71 in the Central Plain of China. Plos One.

[16] Zhang Y (2010). Molecular Evidence of Persistent Epidemic and Evolution of Subgenotype B1 Coxsackievirus A16-Associated Hand, Foot, and Mouth Disease in China. J Clin Microbiol.

[17] Tan X, Haung X, Zhu S (2011). The Persistent Circulation of Enterovirus 71 in People’ s Republic of China: Causing Emerging Nationwide Epidemics Since 2008. Plos One.

[18] Tian H (2017). Epidemiological and aetiological characteristics of hand, foot, and mouth disease in Shijiazhuang City, Hebei province, China, 2009–2012. Plos One.

[19] Staring J (2018). KREMEN1 Is a Host Entry Receptor for a Major Group of Enteroviruses. Cell Host Microbe.

[20] Tian H (2014). Prevalence of multiple enteroviruses associated with hand, foot, and mouth disease in Shijiazhuang City, Hebei province, China: outbreaks of coxsackieviruses a10 and b3. Plos One.

[21] Chen J, Zhang R, Ou X, Chen F, Sun B (2014). The role of enterovirus 71 and coxsackievirus A strains in a large outbreak of hand, foot, and mouth disease in 2012 in Changsha, China. Int J Infect Dis.

[22] Wang, J. *et al*. Epidemiological characteristics of hand, foot, and mouth disease in Shandong, China, 2009–2016. *Sci Rep-Uk* **7** (2017). 10.1038/s41598-017-09196-z PMC5567189 28827733

[23] Ma J (2015). Genetic characteristics of VP1 region of coxsackievirus A10 strains isolated from hand foot and mouth disease patients in Ningxia Hui Autonomous Region, 2013. Zhonghua Liu Xing Bing Xue Za Zhi.

[24] Chen W (2016). Molecular epidemiology of hand-foot-mouth disease associated pathogen Coxsackievirus A10 identified in Fujian province, 2011–2014. Zhonghua Liu Xing Bing Xue Za Zhi.

[25] Tan X (2015). Molecular epidemiology of coxsackievirus A6 associated with outbreaks of hand, foot, and mouth disease in Tianjin, China, in 2013. Arch Virol.

[26] Han JF (2014). Hand, foot, and mouth disease outbreak caused by coxsackievirus A6, China, 2013. J Infect.

[27] Song Y (2017). Persistent circulation of Coxsackievirus A6 of genotype D3 in mainland of China between 2008 and 2015. Sci Rep.

[28] Ang LW (2009). Epidemiology and control of hand, foot and mouth disease in Singapore, 2001–2007. Ann Acad Med Singapore.

[29] Zhang J (2011). Characterization of hand, foot, and mouth disease in China between 2008 and 2009. Biomed Environ Sci.

[30] Zhuang Z (2015). Epidemiological Research on Hand, Foot, and Mouth Disease in Mainland China. Viruses.

[31] Xing W (2014). Hand, foot, and mouth disease in China, 2008–12: an epidemiological study. Lancet Infect Dis.

[32] Xu, M. *et al*. Enterovirus genotypes causing hand foot and mouth disease in Shanghai, China: a molecular epidemiological analysis. **13**, 489 (2013). 10.1186/1471-2334-13-489 PMC4015791 24148902

[33] Dalldorf, G. The coxsackie virus group. *Ann N Y Acad Sci* 583 (1953). 10.1111/j.1749-6632.1953.tb30251.x 13139263

[34] Blomqvist S, Paananen A, Savolainen-Kopra C, Hovi T, Roivainen M (2008). Eight years of experience with molecular identification of human enteroviruses. J Clin Microbiol.

[35] Khetsuriani N, Lamonte-Fowlkes A, Oberst S, Pallansch MA (2006). Enterovirus surveillance–United States, 1970–2005. MMWR Surveill Summ.

[36] Nairn C, Clements GB (1999). A study of enterovirus isolations in Glasgow from 1977 to 1997. J Med Virol.

[37] Ortner B (2009). Epidemiology of enterovirus types causing neurological disease in Austria 1999–2007: detection of clusters of echovirus 30 and enterovirus 71 and analysis of prevalent genotypes. J Med Virol.

[38] Prevention, C. F. D. C., N Enterovirus and Human Parechovirus Surveillance — United States, 2006–2008. *MMWR Morbidity and Mortality Weekly Report* **59** (2010). 21150865

[39] Li JX (2016). Two-year efficacy and immunogenicity of Sinovac Enterovirus 71 vaccine against hand, foot and mouth disease in children. Expert Rev Vaccines.

[40] Zhu FC (2013). Efficacy, safety, and immunology of an inactivated alum-adjuvant enterovirus 71 vaccine in children in China: a multicentre, randomised, double-blind, placebo-controlled, phase 3 trial. Lancet.

[41] Zhu F (2014). Efficacy, safety, and immunogenicity of an enterovirus 71 vaccine in China. N Engl J Med.

[42] Li Y (2017). Epidemiological and genetic analysis concerning the non-enterovirus 71 and non-coxsackievirus A16 causative agents related to hand, foot and mouth disease in Anyang city, Henan Province, China, from 2011 to 2015. J Med Virol.

[43] Zhang Y (2012). Single endemic genotype of measles virus continuously circulating in China for at least 16 years. Plos One.

[44] Zhang Y (2007). Molecular epidemiology of measles viruses in China, 1995–2003. Virol J.

[45] Song J (2017). Emergence of BA9 genotype of human respiratory syncytial virus subgroup B in China from 2006 to 2014. Sci Rep.

[46] Song J (2017). Emergence of ON1 genotype of human respiratory syncytial virus subgroup A in China between 2011 and 2015. Sci Rep.

