# Supplementary material for: The emerging sub-genotype C2 of CoxsackievirusA10 Associated with Hand, Foot and Mouth Disease extensively circulating in mainland of China
Source: Sci Rep. 2018 Sep 6;8:13357. doi: 10.1038/s41598-018-31616-x (PMC6127217; doi:10.1038/s41598-018-31616-x)
Supplement: Supplementary file 1 — Geographic distribution of 1044 Chinese CV-A10 strains over time [file 41598_2018_31616_MOESM1_ESM.pdf]

The emerging sub-genotype C2 of *Coxsackievirus*A10 Associated with Hand， Foot and Mouth Disease extensively circulating in mainland of China

Tianjiao Ji[1], Yue Guo[1], Wei huang[2], Yong Shi[3], Yi Xu[4], Wenbin Tong[5], Wenqing Yao[6], Zhaolin Tan[7], Hanri Zeng[8], Jiangtao Ma[9], Hua Zhao[10], Taoli Han[1], Yong Zhang[1], Dongmei Yan[1], Qian Yang[1], Shuangli Zhu[1], Yan Zhang[1]* and Wenbo Xu[1]*

Table 1. Geographic distribution of 1044 Chinese CV-A10 strains over time

Year	North China	East China	Central China	South China	Southwest China	Northwest China	Northeast China	Total
2004	0	1	0	0	0	0	0	1
2006	0	1	0	0	0	0	0	1
2008	0	4	0	2	0	0	0	6
2009	5	15	6	15	0	0	0	41
2010	18	13	8	21	2	3	3	68
2011	0	8	3	16	3	2	0	32
2012	36	0	20	10	13	10	22	111
2013	9	39	21	22	10	8	1	110
2014	8	58	21	2	44	45	25	203
2015	8	64	4	13	26	10	3	128
2016	59	137	17	9	34	11	76	343
Total	143	340	100	110	132	89	130	1044

Note:

North China: Tianjin, Beijing, Hebei, Inner Mongolia, Shanxi

East China: Fujian, Anhui, Jiangsu, Zhejiang, Shanghai, Shandong, Jiangxi, Taiwan

Central China: Henan, Hubei, Hunan

South China: Guangdong, Guangxi, Hainan, Hong Kong, Macao

Southwest China: Sichuan, Chongqing, Yunnan, Guizhou, Tibet

Northwest China: Shaanxi, Ningxia, Qinghai, Gansu, Xinjiang

Northeast China: Liaoning, Jilin, Heilongjiang
