# Supplementary material for: The emerging sub-genotype C2 of CoxsackievirusA10 Associated with Hand, Foot and Mouth Disease extensively circulating in mainland of China
Source: Sci Rep. 2018 Sep 6;8:13357. doi: 10.1038/s41598-018-31616-x (PMC6127217; doi:10.1038/s41598-018-31616-x)
Supplement: Supplementary file 2 — Reference strains of CV-A10 used for phylogenetic analysis from GenBank [file 41598_2018_31616_MOESM2_ESM.pdf]

The emerging sub-genotype C2 of *Coxsackievirus*A10 Associated with Hand，Foot and Mouth Disease extensively circulating in mainland of China

Tianjiao Ji[1], Yue Guo[1], Wei huang[2], Yong Shi[3], Yi Xu[4], Wenbin Tong[5], Wenqing Yao[6], Zhaolin Tan[7], Hanri Zeng[8], Jiangtao Ma[9], Hua Zhao[10], Taoli Han[1], Yong Zhang[1], Dongmei Yan[1], Qian Yang[1], Shuangli Zhu[1], Yan Zhang[1]* and Wenbo Xu[1]*

Table2. Reference strains of CV-A10 used for phylogenetic analysis from GenBank

Strain name	GenBank number	Country	Year	Strain name	GenBank number	Country	Year
AY421767-Kowalik-complete genome	*AY421767*	*America*	*1950*	*KC867030-Shenzhen/GD/CHN-2009*	*KC867030*	*China*	*2009*
AF081300-Kowalik-VP1	*AF081300*	*America*	*1950*	*KC867028-Shenzhen/GD/CHN-2009*	*KC867028*	*China*	*2009*
KX430809-VNM-2014	*KX430809*	*Viet Nam*	*2014*	*KC867026-Shenzhen/GD/CHN-2009*	*KC867026*	*China*	*2009*
KX430807-VNM-2014	*KX430807*	*Viet Nam*	*2014*	*JX154977-Shenzhen/GD/CHN-2010*	*JX154977*	*China*	*2010*
KX430806-VNM-2014	*KX430806*	*Viet Nam*	*2014*	*JX154976-Shenzhen/GD/CHN-2010*	*JX154976*	*China*	*2010*
KF412913-India-2005	*KF412913*	*India*	*2005*	*JX154975-Shenzhen/GD/CHN-2010*	*JX154975*	*China*	*2010*
KF413053-India-2005	*KF413053*	*India*	*2005*	*JX154973-Shenzhen/GD/CHN-2010*	*JX154973*	*China*	*2010*
JN255588-African-2006	*JN255588*	*African*	*2006*	*JX154971-Shenzhen/GD/CHN-2010*	*JX154971*	*China*	*2010*
JX307651-Cameroon-2008	*JX307651*	*Cameroon*	*2008*	*JX154970-Shenzhen/GD/CHN-2010*	*JX154970*	*China*	*2010*
JN639891-Jiangsu/CHN-2011	*JN639891*	*China*	*2011*	*LC120874-YN/CHN-2014*	*LC120874*	*China*	*2014*
JN639887-Jiangsu/CHN-2011	*JN639887*	*China*	*2011*	*LC013468-YN/CHN/2014*	*LC013468*	*China*	*2014*
JN639886-Jiangsu/CHN-2011	*JN639886*	*China*	*2011*	*LC013457-YN/CHN/2014*	*LC013457*	*China*	*2014*
FR796490-SP-2008	*FR796490*	*Spain*	*2008*	*LC013418-YN/CHN/2014*	*LC013418*	*China*	*2014*
FR796483-SP-2008	*FR796483*	*Spain*	*2008*	*LC013415-YN/CHN/2014*	*LC013415*	*China*	*2014*
FR796479-SP-2008	*FR796479*	*Spain*	*2008*	*LC167417-YN/CHN/2016*	*LC167417*	*China*	*2016*
HE572990-FRA-2010	*HE572990*	*France*	*2010*	*LC167416-YN/CHN/2016*	*LC167416*	*China*	*2016*
HE572969-FRA-2010	*HE572969*	*France*	*2010*	*LC167415-YN/CHN/2016*	*LC167415*	*China*	*2016*
HE572957-FRA-2010	*HE572957*	*France*	*2010*	*KP005987-Shenzhen/GD/CHN-2013*	*KP005987*	*China*	*2013*
HE572948-FRA-2010	*HE572948*	*France*	*2010*	*KP005986-Shenzhen/GD/CHN-2013*	*KP005986*	*China*	*2013*
KR185980-Rusia-2013	*KR185980*	*Russia*	*2013*	*KP005985-Shenzhen/GD/CHN-2013*	*KP005985*	*China*	*2013*
KP289401-wenzhou/ZJ/CHN/2013	*KP289401*	*China*	*2013*	*KP009581-FY/AH/CHN/2013*	*KP009581*	*China*	*2013*
KP289402-wenzhou/ZJ/CHN/2013	*KP289402*	*China*	*2013*	*KP009580-FY/AH/CHN/2013*	*KP009580*	*China*	*2013*
KP289403-wenzhou/ZJ/CHN/2013	*KP289403*	*China*	*2013*	*KP009578-FY/AH/CHN/2013*	*KP009578*	*China*	*2013*
KP289405-wenzhou/ZJ/CHN/2013	*KP289405*	*China*	*2013*	*KP009577-FY/AH/CHN/2013*	*KP009577*	*China*	*2013*
KP289407-wenzhou/ZJ/CHN/2013	*KP289407*	*China*	*2013*	*KC867022-Shenzhen/GD/CHN-2012*	*KC867022*	*China*	*2012*
KP289408-wenzhou/ZJ/CHN/2013	*KP289408*	*China*	*2013*	*KC867020-Shenzhen/GD/CHN-2012*	*KC867020*	*China*	*2012*
KP289410-wenzhou/ZJ/CHN/2013	*KP289410*	*China*	*2013*	*KC867019-Shenzhen/GD/CHN-2008*	*KC867019*	*China*	*2008*
KJ641623-Shenzhen/GD/CHN/2013	*KJ641623*	*China*	*2013*	*KC867018-Shenzhen/GD/CHN-2008*	*KC867018*	*China*	*2008*
HQ728262-SD/CHN/2009	*HQ728262*	*China*	*2009*	*JX473456-Shenzhen/GD/CHN-2011*	*JX473456*	*China*	*2011*
KF246675-HeB/CHN/2010	*KF246675*	*China*	*2010*	*JX473455-Shenzhen/GD/CHN-2011*	*JX473455*	*China*	*2011*
KF246671-HeB/CHN/2010	*KF246671*	*China*	*2010*	*JX473454-Shenzhen/GD/CHN-2011*	*JX473454*	*China*	*2011*
KF246668-HeB/CHN/2012	*KF246668*	*China*	*2012*	*JX473453-Shenzhen/GD/CHN-2011*	*JX473453*	*China*	*2011*

KF246665-HeB/CHN/2012	*KF246665*	*China*	*2012*	*JX473452-Shenzhen/GD/CHN-2011*	*JX473452*	*China*	*2011*
KF246663-HeB/CHN/2012	*KF246663*	*China*	*2012*	*JX473451-Shenzhen/GD/CHN-2011*	*JX473451*	*China*	*2011*
GU947787-SD/CHN/2009	*GU947787*	*China*	*2009*	*JX473450-Shenzhen/GD/CHN-2011*	*JX473450*	*China*	*2011*
GU947776-SD/CHN/2009	*GU947776*	*China*	*2009*	*JX473449-Shenzhen/GD/CHN-2011*	*JX473449*	*China*	*2011*
GU947774-SD/CHN/2009	*GU947774*	*China*	*2009*	*MF422532-TW-2008*	*MF422532*	*China*	*2008*
GQ214176-SD/CHN/2004	*GQ214176*	*China*	*2004*	*MF422531-TW-2008*	*MF422533*	*China*	*2008*
KJ784513-HuN/CHN/2012	*KJ784513*	*China*	*2012*	*JN896804-TW/09*	*JN896804*	*China*	*2009*
KJ784512-HuN/CHN/2012	*KJ784512*	*China*	*2012*	*JN896805-TW/09*	*JN896805*	*China*	*2009*
KJ784511-HuN/CHN/2012	*KJ784511*	*China*	*2012*	*JN896784-TW/08*	*JN896784*	*China*	*2008*
KJ156358-HuN/CHN/2012	*KJ156358*	*China*	*2012*	*JN896799-TW/09*	*JN896799*	*China*	*2009*
KJ156357-HuN/CHN/2012	*KJ156357*	*China*	*2012*	*JN896779-TW/08*	*JN896779*	*China*	*2008*
KJ156356-HuN/CHN/2012	*KJ156356*	*China*	*2012*	*KM816543-TW/2011*	*KM816543*	*China*	*2011*
KJ156355-HuN/CHN/2012	*KJ156355*	*China*	*2012*	*KM816549-TW/2011*	*KM816549*	*China*	*2011*
KJ156354-HuN/CHN/2012	*KJ156354*	*China*	*2012*	*KM816550-TW/2012*	*KM816550*	*China*	*2012*
KF150149-SD/CHN/2010	*KF150149*	*China*	*2010*	*KM816537-TW/2011*	*KM816537*	*China*	*2011*
GQ214177-SD/CHN/2006	*GQ214177*	*China*	*2006*	*KM816538-TW/2011*	*KM816538*	*China*	*2011*
GQ214175-SD/CHN/2008	*GQ214175*	*China*	*2008*	*HQ844648-YN-2008*	*HQ844648*	*China*	*2008*
GQ214174-SD/CHN/2008	*GQ214174*	*China*	*2008*	*KC834877-SH/CHN/2011*	*KC834877*	*China*	*2011*
GQ214173-SD/CHN/2008	*GQ214173*	*China*	*2008*	*GU248502-FIN08*	*GU248502*	*Finland*	*2008*
GQ214172-SD/CHN/2008	*GQ214172*	*China*	*2008*	*GU248491-EGY07*	*GU248491*	*Egypt*	*2007*
KU885571-HeN/CHN/2014	*KU885571*	*China*	*2014*	*GU248490-Russia-01*	*GU248490*	*Russia*	*2001*
KU885570-HeN/CHN/2014	*KU885570*	*China*	*2014*	*GU248489-FIN99*	*GU248489*	*Finland*	*1999*
KU885567-HeN/CHN/2014	*KU885567*	*China*	*2014*	*GU248486-Slovakia-07*	*GU248486*	*Slovakia*	*2007*
KU885566-HeN/CHN/2014	*KU885566*	*China*	*2014*	*GU248485-Slovakia-02*	*GU248485*	*Slovakia*	*2002*
KU885563-HeN/CHN/2014	*KU885563*	*China*	*2014*	*JN034210-GERMANY2006*	*JN034210*	*Germany*	*2006*
KU885562-HeN/CHN/2014	*KU885562*	*China*	*2014*	*JN034211-GERMANY2001*	*JN034211*	*Germany*	*2001*
KU885560-HeN/CHN/2014	*KU885560*	*China*	*2014*	*GQ214172-SD/CHN/2008*	*GQ214172*	*China*	*2008*
KU885558-HeN/CHN/2014	*KU885558*	*China*	*2014*	*KX304013-Italy/2014*	*KX304013*	*Italy*	*2014*
KU885553-HeN/CHN/2013	*KU885553*	*China*	*2013*	*KC954150-Italy/2012*	*KC954150*	*Italy*	*2012*
KU885555-HeN/CHN/2013	*KU885555*	*China*	*2013*	*KF006263-Italy/2012*	*KF006263*	*Italy*	*2012*
KU885552-HeN/CHN/2012	*KU885552*	*China*	*2012*	*KY762310-USA/AZ/2016*	*KY762310*	*USA*	*2016*
KU885551-HeN/CHN/2012	*KU885551*	*China*	*2012*	*GQ914903-Georgia-2003*	*GQ914903*	*Georgia*	*2003*
KU885550-HeN/CHN/2012	*KU885550*	*China*	*2012*	*JX538121-Bangladesh/2007*	*JX538121*	*Bangladesh*	*2007*
KU885549-HeN/CHN/2012	*KU885549*	*China*	*2012*	*JX538055-Bangladesh/2007*	*JX538055*	*Bangladesh*	*2007*
KU885548-HeN/CHN/2012	*KU885548*	*China*	*2012*	*AB598060-YN/CHN/2010*	*AB598060*	*China*	*2010*
KU885547-HeN/CHN/2012	*KU885547*	*China*	*2012*	*AB902817-Okinawa/Japan/2011*	*AB902817*	*Japan*	*2011*
KU885543-HeN/CHN/2012	*KU885543*	*China*	*2012*	*AB162727-Kanagawa/Japan/2003*	*AB162727*	*Japan*	*2003*
KU885541-HeN/CHN/2011	*KU885541*	*China*	*2011*	*AB162730-Kanagawa/Japan/2003*	*AB162730*	*Japan*	*2003*
KC867044-Shenzhen/GD/CHN-2012	*KC867044*	*China*	*2012*	*AB119638-Hiroshima/JP/00*	*AB119638*	*Japan*	*2000*
KC867043-Shenzhen/GD/CHN-2012	*KC867043*	*China*	*2012*	*AB119639-Hiroshima.JP/01*	*AB119639*	*Japan*	*2001*
KC867042-Shenzhen/GD/CHN-2012	*KC867042*	*China*	*2012*	*AB119640-Hiroshima.JP/03*	*AB119640*	*Japan*	*2003*
KC867040-Shenzhen/GD/CHN-2012	*KC867040*	*China*	*2012*	*AB162728-Kanagawa/2003*	*AB162728*	*Japan*	*2003*
KC867038-Shenzhen/GD/CHN-2012	*KC867038*	*China*	*2012*	*AB167986-Fukuoka/jp/03*	*AB167986*	*Japan*	*2003*
KC867035-Shenzhen/GD/CHN-2012	*KC867035*	*China*	*2012*	*LC124103-Sapporo-2011Jul*	*LC124103*	*Japan*	*2011*

KC867034-Shenzhen/GD/CHN-2009	*KC867034*	*China*	*2009*	*LC124105-Fukuoka-2011Jul*	*LC124105*	*Japan*	*2011*
KC867032-Shenzhen/GD/CHN-2009	*KC867032*	*China*	*2009*	*LC124117-Sapporo-2015Aug*	*LC124117*	*Japan*	*2015*
KC867031-Shenzhen/GD/CHN-2009	*KC867031*	*China*	*2009*				
