# Supplementary material for: The emerging sub-genotype C2 of CoxsackievirusA10 Associated with Hand, Foot and Mouth Disease extensively circulating in mainland of China
Source: Sci Rep. 2018 Sep 6;8:13357. doi: 10.1038/s41598-018-31616-x (PMC6127217; doi:10.1038/s41598-018-31616-x)
Supplement: Supplementary file 3 — The locality and time distribution of the CV-A10 VP1 sequences involved in this study [file 41598_2018_31616_MOESM3_ESM.pdf]

The emerging sub-genotype C2 of *Coxsackievirus*A10 Associated with Hand, Foot and Mouth Disease extensively circulating in mainland of China

Tianjiao Ji[1], Yue Guo[1], Wei huang[2], Yong Shi[3], Yi Xu[4], Wenbin Tong[5], Wenqing Yao[6], Zhaolin Tan[7], Hanri Zeng[8], Jiangtao Ma[9], Hua Zhao[10], Taoli Han[1], Yong Zhang[1], Dongmei Yan[1], Qian Yang[1], Shuangli Zhu[1], Yan Zhang[1]* and Wenbo Xu[1]*

Table 3. The locality and time distribution of the CV-A10 VP1 sequences involved in this study

Strains name	Provinces	Isolated years	Disease Type	GenBank accession No.
AH16-26-CA10	*Anhui*	*2016*	*mild case*	*MG838781*
AH16-36-CA10	*Anhui*	*2016*	*mild case*	*MG838782*
BJ14-50-CA10	*Beijing*	*2014*	*mild case*	*MG838783*
BJ14-110-CA10	*Beijing*	*2014*	*mild case*	*MG838784*
CQ15-27-CVA10	*Chongqing*	*2015*	*mild case*	*MG838785*
CQ16-33	*Chongqing*	*2016*	*mild case*	*MG838786*
GD13-32-CVA10	*Guangdong*	*2013*	*mild case*	*MG838787*
GD13-35-CVA10	*Guangdong*	*2013*	*mild case*	*MG838788*
GD14-1-CA10	*Guangdong*	*2014*	*mild case*	*MG838789*
GD14-32	*Guangdong*	*2014*	*severe disease*	*MG838790*
GD15-57-CA10	*Guangdong*	*2015*	*mild case*	*MG838791*
GD15-65-CA10	*Guangdong*	*2015*	*severe disease*	*MG838792*
GD15-69-CA10	*Guangdong*	*2015*	*mild case*	*MG838793*
GD15-71-CA10	*Guangdong*	*2015*	*mild case*	*MG838794*
GD16-20	*Guangdong*	*2016*	*mild case*	*MG838795*
GS12-64	*Gansu*	*2012*	*mild case*	*MG838796*
GS14-36	*Gansu*	*2014*	*mild case*	*MG838797*
GS14-69	*Gansu*	*2014*	*mild case*	*MG838798*
GS15-43	*Gansu*	*2015*	*severe disease*	*MG838799*
GS15-51	*Gansu*	*2015*	*mild case*	*MG838800*
HeB-2009-36	*Hebei*	*2009*	*mild case*	*MG838801*
HeB-2009-52	*Hebei*	*2009*	*mild case*	*MG838802*
HeB2012-117	*Hebei*	*2012*	*mild case*	*MG838803*
HeB2013-7	*Hebei*	*2013*	*severe disease*	*MG838804*
HeB2013-8	*Hebei*	*2013*	*severe disease*	*MG838805*
HeB2013-61	*Hebei*	*2013*	*severe disease*	*MG838806*
HeB2015-63	*Hebei*	*2015*	*mild case*	*MG838807*
HeN16-8-CA10-VP1	*Henan*	*2016*	*mild case*	*MG838808*
HeN16-17-CA10	*Henan*	*2016*	*mild case*	*MG838809*
HeN16-26-CA10-VP1	*Henan*	*2016*	*mild case*	*MG838810*
HeN16-59-CA10-VP1	*Henan*	*2016*	*mild case*	*MG838811*
HuN11-9	*Hunan*	*2011*	*severe disease*	*MG838812*
HuN13-3	*Hunan*	*2013*	*mild case*	*MG838813*

HuN14-57	*Hunan*	*2014*	*severe disease*	*MG838814*
HuN15-2-CA10	*Hunan*	*2015*	*mild case*	*MG838815*
HuN16-33-CA10	*Hunan*	*2016*	*severe disease*	*MG838816*
JS14-38-CV-A10	*Jiangsu*	*2014*	*mild case*	*MG838817*
JS15-104	*Jiangsu*	*2015*	*severe disease*	*MG838818*
JX13-47	*Jiangxi*	*2013*	*mild case*	*MG838819*
JX13-66	*Jiangxi*	*2013*	*severe disease*	*MG838820*
JX13-99	*Jiangxi*	*2013*	*mild case*	*MG838821*
JX13-101	*Jiangxi*	*2013*	*mild case*	*MG838822*
JX14-19-CA10	*Jiangxi*	*2014*	*mild case*	*MG838823*
JX14-28-CA10	*Jiangxi*	*2014*	*mild case*	*MG838824*
JX14-38-CA10	*Jiangxi*	*2014*	*mild case*	*MG838825*
JX14-128-CA10	*Jiangxi*	*2014*	*severe disease*	*MG838826*
JX15-50	*Jiangxi*	*2015*	*mild case*	*MG838827*
JX15-94	*Jiangxi*	*2015*	*severe disease*	*MG838828*
JX15-97	*Jiangxi*	*2015*	*severe disease*	*MG838829*
JX16-97-CA10-VP1	*Jiangxi*	*2016*	*mild case*	*MG838830*
LN14-108	*Liaoning*	*2014*	*mild case*	*MG838831*
LN14-147	*Liaoning*	*2014*	*severe disease*	*MG838832*
LN14-229	*Liaoning*	*2014*	*mild case*	*MG838833*
LN14-234	*Liaoning*	*2014*	*mild case*	*MG838834*
LN15-104-CA10	*Liaoning*	*2015*	*mild case*	*MG838835*
LN16-13-CA10	*Liaoning*	*2016*	*mild case*	*MG838836*
LN16-34-CA10	*Liaoning*	*2016*	*mild case*	*MG838837*
NX14-61	*Ningxia*	*2014*	*mild case*	*MG838838*
NX16-86	*Ningxia*	*2016*	*mild case*	*MG838839*
QH14-1-CA10	*Qinghai*	*2014*	*mild case*	*MG838840*
QH14-25-CA10	*Qinghai*	*2014*	*mild case*	*MG838841*
QH14-94-CA10	*Qinghai*	*2014*	*mild case*	*MG838842*
QH14-95-CA10	*Qinghai*	*2014*	*mild case*	*MG838843*
QH16-24-CA10	*Qinghai*	*2016*	*mild case*	*MG838844*
SaX13-11	*Shaanxi*	*2013*	*severe disease*	*MG838845*
Sax13-68-RD-CVA10	*Shaanxi*	*2013*	*mild case*	*MG838846*
SaX14-7-CV-A10	*Shaanxi*	*2014*	*mild case*	*MG838847*
SaX14-9-CV-A10	*Shaanxi*	*2014*	*mild case*	*MG838848*
SaX14-13-CV-A10	*Shaanxi*	*2014*	*mild case*	*MG838849*
SaX14-19-CV-A10	*Shaanxi*	*2014*	*severe disease*	*MG838850*
SaX14-21	*Shaanxi*	*2014*	*mild case*	*MG838851*
SaX14-29-CV-A10	*Shaanxi*	*2014*	*mild case*	*MG838852*
SaX14-84-CV-A10	*Shaanxi*	*2014*	*severe disease*	*MG838853*
SaX16-50	*Shaanxi*	*2016*	*severe disease*	*MG838854*
SaX16-98	*Shaanxi*	*2016*	*severe disease*	*MG838855*
SC14-49-CA10	*Sichuan*	*2014*	*mild case*	*MG838856*
SC14-66-CA10	*Sichuan*	*2014*	*mild case*	*MG838857*

SC14-77-CA10	*Sichuan*	*2014*	*mild case*	*MG838858*
SC15-43-CA10	*Sichuan*	*2015*	*mild case*	*MG838859*
SC15-91-CA10	*Sichuan*	*2015*	*mild case*	*MG838860*
SC16-106-CA10	*Sichuan*	*2016*	*mild case*	*MG838861*
SD-2013-246	*Shandong*	*2013*	*mild case*	*MG838862*
SD-2014-15	*Shandong*	*2014*	*mild case*	*MG838863*
SD-2014-57	*Shandong*	*2014*	*mild case*	*MG838864*
SD-2014-65	*Shandong*	*2014*	*mild case*	*MG838865*
SD-2014-102	*Shandong*	*2014*	*mild case*	*MG838866*
SD-2015-64	*Shandong*	*2015*	*mild case*	*MG838867*
SD2015-68-CA10-VP1	*Shandong*	*2015*	*severe disease*	*MG838868*
SD-2015-175	*Shandong*	*2015*	*mild case*	*MG838869*
SX-2010-47	*Shanxi*	*2010*	*mild case*	*MG838870*
SX-2010-48	*Shanxi*	*2010*	*mild case*	*MG838871*
TJ13-35-RD-CVA10	*Tianjin*	*2013*	*mild case*	*MG838872*
TJ15-70-YZ	*Tianjin*	*2015*	*mild case*	*MG838873*
TJ15-99	*Tianjin*	*2015*	*mild case*	*MG838874*
TJ16-17-CVA10	*Tianjin*	*2016*	*mild case*	*MG838875*
TJ16-36-CVA10	*Tianjin*	*2016*	*mild case*	*MG838876*
TJ16-62-CVA10	*Tianjin*	*2016*	*mild case*	*MG838877*
XJ13-35	*Xinjiang*	*2013*	*mild case*	*MG838878*
XJ14-64	*Xinjiang*	*2014*	*mild case*	*MG838879*
XJ14-66	*Xinjiang*	*2014*	*mild case*	*MG838880*
YN14-94	*Yunnan*	*2014*	*severe disease*	*MG838881*
YN15-15CV10	*Yunnan*	*2015*	*mild case*	*MG838882*
YN15-138-CV10	*Yunnan*	*2015*	*mild case*	*MG838883*
YN15-184-CV10	*Yunnan*	*2015*	*mild case*	*MG838884*
CQ11-61	*Chongqing*	*2011*	*mild case*	*KF999730*
CQ11-63	*Chongqing*	*2011*	*mild case*	*KF999731*
CQ11-64	*Chongqing*	*2011*	*mild case*	*KF999732*
CQ12-1	*Chongqing*	*2012*	*mild case*	*KF999733*
CQ12-50	*Chongqing*	*2012*	*mild case*	*KF999738*
CQ12-52	*Chongqing*	*2012*	*mild case*	*KF999739*
HaN10-189	*Hainan*	*2010*	*severe disease*	*KF999740*
HaN10-215	*Hainan*	*2010*	*mild case*	*KF999742*
HLJ10-16	*Heilongjiang*	*2010*	*mild case*	*KF999744*
HLJ10-19	*Heilongjiang*	*2010*	*mild case*	*KF999745*
HuN09-19	*Hunan*	*2009*	*mild case*	*KF999746*
HuN09-22	*Hunan*	*2009*	*mild case*	*KF999747*
HuN09-23	*Hunan*	*2009*	*mild case*	*KF999748*
HuN09-51	*Hunan*	*2009*	*mild case*	*KF999750*
HuN10-105	*Hunan*	*2010*	*mild case*	*KF999751*
HuN10-109	*Hunan*	*2010*	*severe disease*	*KF999752*
HuN10-110	*Hunan*	*2010*	*severe disease*	*KF999753*

HuN10-111	*Hunan*	*2010*	*severe disease*	*KF999754*
HuN10-113	*Hunan*	*2010*	*severe disease*	*KF999755*
HuN10-116	*Hunan*	*2010*	*severe disease*	*KF999756*
HuN10-122	*Hunan*	*2010*	*mild case*	*KF999757*
HuN10-123	*Hunan*	*2010*	*severe disease*	*KF999758*
JL12-2	*Jilin*	*2012*	*mild case*	*KF999759*
JL12-34	*Jilin*	*2012*	*mild case*	*KF999761*
JL12-39	*Jilin*	*2012*	*mild case*	*KF999762*
JL12-51	*Jilin*	*2012*	*mild case*	*KF999764*
JX11-45	*Jiangxi*	*2011*	*mild case*	*KF999768*
JX11-120	*Jiangxi*	*2011*	*mild case*	*KF999771*
JX11-131	*Jiangxi*	*2011*	*mild case*	*KF999772*
JX11-156	*Jiangxi*	*2011*	*mild case*	*KF999773*
LN10-44	*Liaoning*	*2010*	*mild case*	*KF999774*
LN12-92	*Liaoning*	*2012*	*mild case*	*KF999775*
LN12-94	*Liaoning*	*2012*	*mild case*	*KF999776*
LN12-97	*Liaoning*	*2012*	*mild case*	*KF999777*
NX10-37	*Ningxia*	*2010*	*severe disease*	*KF999779*
NX10-152	*Ningxia*	*2010*	*mild case*	*KF999780*
NX10-155	*Ningxia*	*2010*	*mild case*	*KF999781*
NX11-145	*Ningxia*	*2011*	*mild case*	*KF999782*
NX11-146	*Ningxia*	*2011*	*mild case*	*KF999783*
NX12-53	*Ningxia*	*2012*	*mild case*	*KF999784*
ZJ10-122	*Zhejiang*	*2010*	*mild case*	*KF999785*
ZJ10-146	*Zhejiang*	*2010*	*mild case*	*KF999786*
